# A Cross-sectional Study of Current Doctors’ Performance in a Modified Version of a Medical School Admission Aptitude Test: The UKCAT

**DOI:** 10.1097/MD.0000000000003506

**Published:** 2016-05-06

**Authors:** James P. Blackmur, Nazir I. Lone, Oliver D. Stone, David J. Webb, Neeraj Dhaun

**Affiliations:** From the University/British Heart Foundation Centre of Research Excellence (JPB, DJW, ND); Usher Institute of Population Health Sciences and Informatics, University of Edinburgh (NIL); and Department of Orthopaedics, Royal Infirmary of Edinburgh (ODS), Edinburgh, UK.

## Abstract

The 2-hour long United Kingdom Clinical Aptitude Test (UKCAT) is used by many universities in the United Kingdom as part of their selection process for undergraduate medical and dentistry degrees. We aimed to compare the performance of senior doctors in primary and secondary care and across a range of specialties, in a modified version of the medical school entrance examination—the *m*UKCAT. Lay people were also included in the study. Despite its widespread use, this is the first study that examines the performance of senior clinicians in the UKCAT.

The study used a prospective cross-sectional design. It used mock questions from the UKCAT website to generate an mUKCAT that was anticipated to take 15 minutes to complete. In all, 167 doctors at consultant, general practitioner (GP), or specialty trainee grade and 26 lay people took part.

The overall mean *m*UKCAT score of all participants was 2486 (69.1%). Of the total cohort, 126 (65.3%) scored above our designated threshold of 2368 and were deemed to have passed the *m*UKCAT. Excluding lay people, 113 (67.7%) of the 167 doctors scored above that threshold. Medical specialty was associated with overall score (*P* = 0.003), with anesthetists/intensive care physicians scoring highest (n = 20, mean score 2660) and GPs scoring lowest (n = 38, mean score 2302). Academics outperformed nonacademics (mean score of academics, n = 44 vs nonacademics, n = 123: 2750 vs 2406; *P* < 0.001). Those clinicians in senior management positions scored lower than those in “standard” roles (mean score of senior management, n = 31 vs standard roles, n = 136: 2332 vs 2534, mean difference 202, 95% confidence interval 67–337, *P* = 0.004).

In the situational judgement section, there was no evidence that specialty was associated with score (*P* = 0.15). Academics exhibited greater situational judgement than their nonacademic colleagues (academics vs nonacademics: 69.8 vs 63.6%; *P* = 0.01).

The majority of senior clinicians passed our *m*UKCAT. Academics and anesthetists were found to be the best performers, with GPs and those in senior management positions performing the worst.

## Abstract 2

Supplemental Digital Content is available in the text

## INTRODUCTION

The United Kingdom Clinical Aptitude Test (UKCAT) is used by many universities in the United Kingdom as part of their selection process for undergraduate medical and dentistry degrees. It was introduced in 2006 to “ensure that candidates selected have the most appropriate mental abilities, attitudes, and professional behavior required for new doctors and dentists to be successful in their clinical careers.”^1^ School students take the UKCAT before applying for undergraduate medicine or dentistry through UCAS, the Universities and Colleges Admissions Service.^2^ The method in which the UKCAT scores are utilized by individual universities varies. Some use the UKCAT score as an absolute cut-off for further consideration for entry into medical school, others take the UKCAT score alongside other measures, and a couple use it to discriminate between candidates scoring the same in other assessment areas.^3^

In the United States (US) and Canada, the Medical College Admission Test (MCAT) is used as a prerequisite for entry into the study of medicine. This covers 4 areas: physical sciences, biological sciences, verbal reasoning, and a writing sample. The UKCAT is 2 hours long and consists of questions across 5 domains—quantitative reasoning (mathematics), verbal reasoning (comprehension testing), abstract reasoning (pattern recognition), decision analysis (code breaking), and situational judgement.^4^ The first 4 sections are marked on the number of correct answers a candidate gives, with no negative marking for incorrect answers. As the number of questions varies between these 4 cognitive sections, it is not possible to directly compare raw scores. These are, therefore, scaled into a shared common range that varies from 300 to 900. A candidate's total UKCAT score, which ranges from 1200 to 3600, is the sum of these 4-scaled scores. The situational judgment section is considered separately to the overall score from the 4 cognitive domains.^4^ Individual universities use the UKCAT scores differently when considering student applications to medicine.^5^

With no curriculum content, study of the UKCAT can give an understanding of the usefulness and consequences of aptitude testing in determining entry to medical school. Whereas some studies have suggested that UKCAT scores correlate with academic performance in the early^6^ and later years^7,8^ of medical school, others have shown these to be of limited predictive value.^9,10^ Whereas the extent to which the UKCAT can predict performance in medical school and widen participation has previously been discussed,^6^ no study to date has examined the performance of qualified doctors, in particular, senior clinicians, in the UKCAT, and questioned its impact on defining the future medical workforce.

Our aim was to assess senior clinicians’ performance in a shortened version of the UKCAT (*m*UKCAT). Overall, we hypothesized that the majority of qualified doctors would not achieve a sufficiently high mark in the *m*UKCAT to be considered for entry into medical school. Additionally, we hypothesized that *m*UKCAT scores would vary amongst specialties; the more senior the clinician, the lower would be their score; academic clinicians would outperform nonacademics; those in senior management positions would outperform their colleagues in standard roles.

## METHODS

As described, candidates are normally allowed 2 hours to complete the real UKCAT (designated *r*UKCAT in this study), which consists of questions across 5 domains. We created an abbreviated version of the *r*UKCAT utilizing mock questions available from the UKCAT website.^11^ Questions from the online mock UKCAT tests were assigned numbers; our modified test (*m*UKCAT) questions were then selected via a random number generator. The length of rubric varies by domain and question. Overall, our study was designed to take approximately 3 minutes per domain. Our *m*UKCAT consisted of 28 questions and was designed to take 15 minutes to complete. This length of time was considered reasonable for working clinicians to give up for the study. A longer test would likely have limited participation. The number of questions per domain was as follows: quantitative reasoning 4, verbal reasoning 4, abstract reasoning 6, decision analysis 3, and situational judgement 11.

The study was prospective and cross-sectional in design. Consultants and senior trainees (specialty trainee 3 [ST3] grade and above) in a range of hospital-based specialties and general practice were invited to take part in the study via e-mail or face-to-face invitation. National Health Service (NHS) trusts and health boards primarily included were NHS Lothian, Fife, Forth Valley, Borders, Tayside, and Imperial College. These were selected due to availability to the authors of regional e-mail distribution lists; from these, participants were targeted to give a range of specialty representatives to the study. We also approached a number of nonmedical professionals. Completed tests were e-mailed or handed back to the authors for marking. Responders were made aware of their score, but did not receive further feedback on the examination. Those who completed the test were encouraged to forward it on to colleagues, friends, and family. By this method, responses were received from 23 different UK NHS regions. Academics were defined as those primarily employed with a university contract and nonacademics as those primarily employed with an NHS contract. Participants were also stratified according to whether they held a designated senior NHS/Deanery management role (eg, clinical director, training program director) or not. Participants were told to take 15 minutes to complete the test, although it was not practical to ask them to undertake the test under examination conditions.

### Validation of the mUKCAT

**FIGURE 1:**
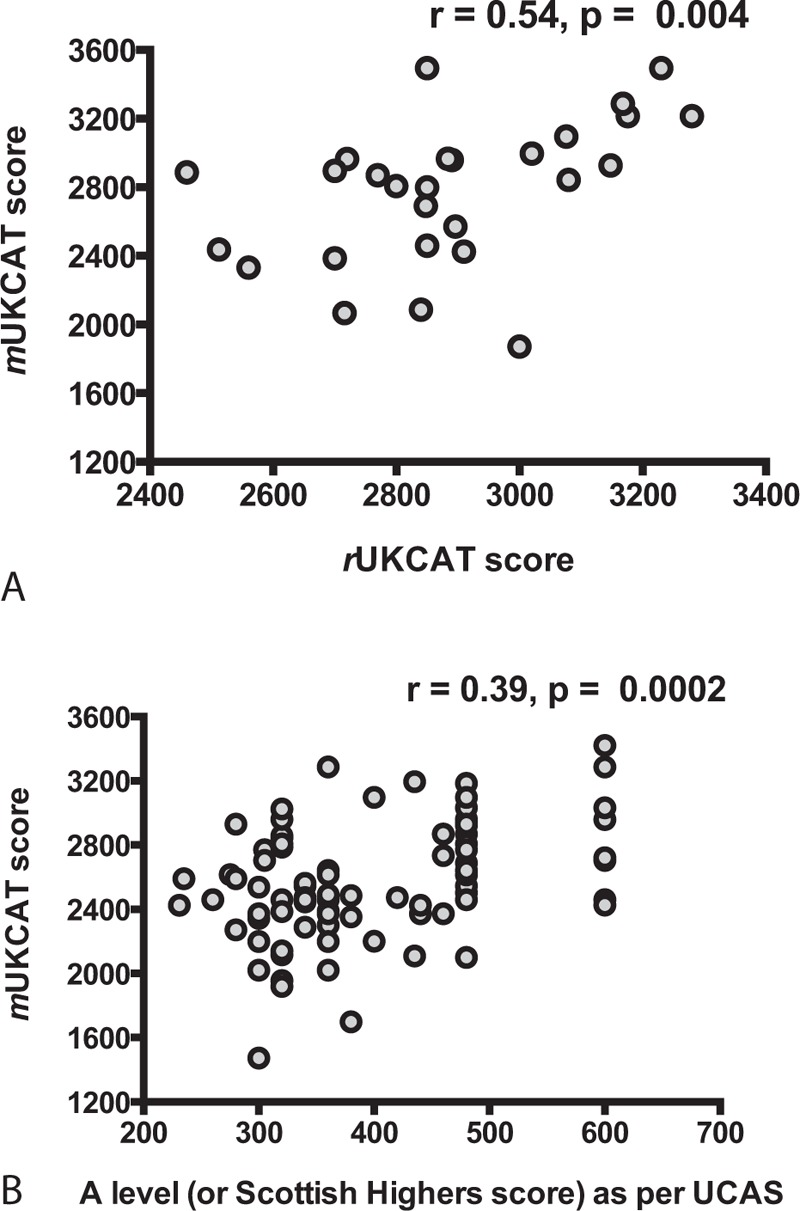
Scatter plots of (A) medical student *r*UKCAT score against *m*UKCAT score and (B) actual A-level (or Scottish Higher) score by UCAS against *m*UKCAT score.

Our *m*UKCAT consisted of mock questions from the UKCAT website.^11^ These are the only official questions freely available to potential applicants, and are “of an equivalent standard to those [a candidate] will encounter in the test.”^12^ We confirmed that our *m*UKCAT test was valid and fit for purpose in 2 ways. First, we asked 27 current medical students to complete our test within 15 minutes and under examinations conditions. These students had previously sat the *r*UKCAT as part of their entrance criteria for medical school. We compared their actual premedical school *r*UKCAT score with that from our *m*UKCAT using Spearman rho correlation coefficient. We found a strong positive correlation between the 2 (*r* = 0.54, *P* = 0.004; Figure 1A). Second, it has been reported there is an association between the score achieved by an individual in the *r*UKCAT and their A-level score.^13^ Thus, we asked those who completed our study to also disclose their A-level scores (or Higher scores for those from Scotland). Ninety senior clinicians provided these data. A-level or higher grades were scored based on the UCAS tariff tables.^14^ We found a moderately strong positive correlation using Spearman rho coefficient between the 2 variables (*r* = 0.39, *P* < 0.001; Figure 1B). However, we recognize that some of these participants sat their A-levels as far back as the 1960s, making this comparison less robust, and given grade inflation over time, this is a less important correlation than that of the medical students.

### Passing the mUKCAT

The *r*UKCAT threshold score for successful application to medical school differs for each university and on each admission cycle, and is dependent on the scores of all those applying. Based on the medical schools that publish their recent successful applicants’ *r*UKCAT scores,^5^ we calculated that for our own test, a minimum score of 2368 (out of 3600, 65.8%) would be required for interview/successful application (see supplementary information). Those scoring above this level were deemed to have “passed” our *m*UKCAT.

### Transformation of Raw mUKCAT Score to Scaled mUKCAT Score

For comparison with the *r*UKCAT, it was important to transform our raw *m*UKCAT scores out of 28 to scaled scores out of 3600. The score for each of the 4 domains was initially transformed so that the denominator was the same as that used in the *r*UKCAT examination (quantitative reasoning 32, verbal reasoning 40, abstract reasoning 50, decision analysis 26; see UKCAT Technical Report, 2013).^15^ In the *r*UKCAT, each domain is transformed to a scaled score, with a minimum value of 300 and a maximum value of 900. These are then summed to give a total score out of 3600. The method used to transform raw scores to scaled scores is not available from UKCAT. Therefore, to transform our test scores from 28 questions to scaled scores out of 3600, we used the reported mean and standard deviation for the national UKCAT examination 2013 to simulate datasets with 1,000,000 observations for raw test scores and scaled scores assuming a beta distribution for each domain. The 2 parameters of the beta distribution were varied until the resulting simulated dataset had identical mean and standard deviation to those reported. We then paired the simulated raw and scaled scores for each domain to create a lookup dataset. The corresponding scaled score could then be drawn for each test score in our study. Hereon, the term “*m*UKCAT score” refers to the scaled total score for the 4 domains: quantitative reasoning, verbal reasoning, abstract reasoning, and decision analysis. The situational judgement section is considered separately to the other 4 domains in the *r*UKCAT, and as it is not scaled, we have reported this score separately as a percentage.

### Statistical Analysis

The current study was powered on the basis of preliminary data that showed a mean score of 2342 ± 336 for general practitioners (GPs) and 2609 ± 314 for physicians. To detect a difference of 10% between physicians and GPs with 90% power and at *P* < 0.05, 32 subjects were required in each group. All analyses were undertaken using Stata 13 (Statacorp, TX) and IBM SPSS version 19 (SPSS, Chicago, IL). Significance was assumed at the 0.05 level. Differences between mean *m*UKCAT score were analyzed using *t* test or analysis of variance (ANOVA). Differences in mean situational judgment score were assessed using Mann–Whitney or Kruskal–Wallis test.

As per the NHS Health Research Authority National Research Ethics Service, the study did not require Ethics Committee approval as it did not involve medicinal products, exposure to ionizing radiation, use of tissue from living or deceased subjects, or individuals lacking capacity.

## RESULTS

One hundred ninety-three individuals responded to the study and completed all 5 sections of our test. This comprised 167 doctors and 26 lay people. One hundred twenty-six (65.3%) of the total cohort scored above our designated threshold of 2368 and were deemed to have passed *m*UKCAT. Excluding lay people, 113 (67.7%) of the 167 doctors scored above that threshold.

### Score Comparison

**FIGURE 2:**
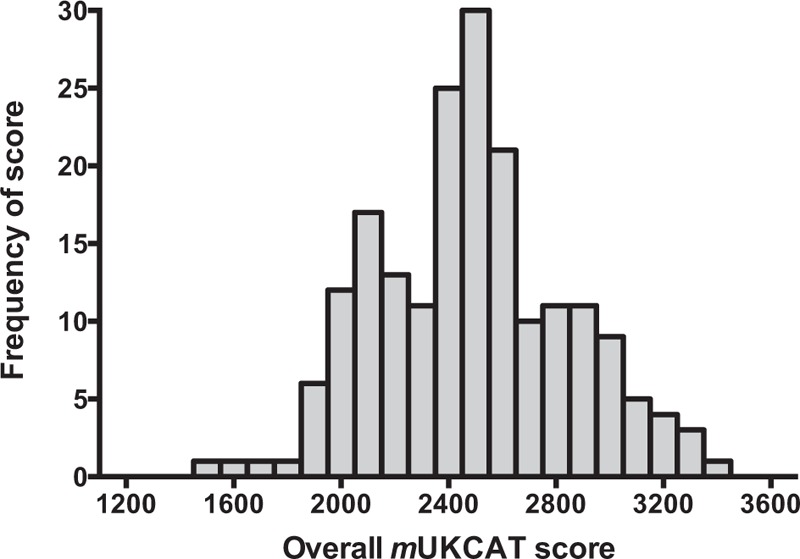
Spread of overall *m*UKCAT score.

**FIGURE 3:**
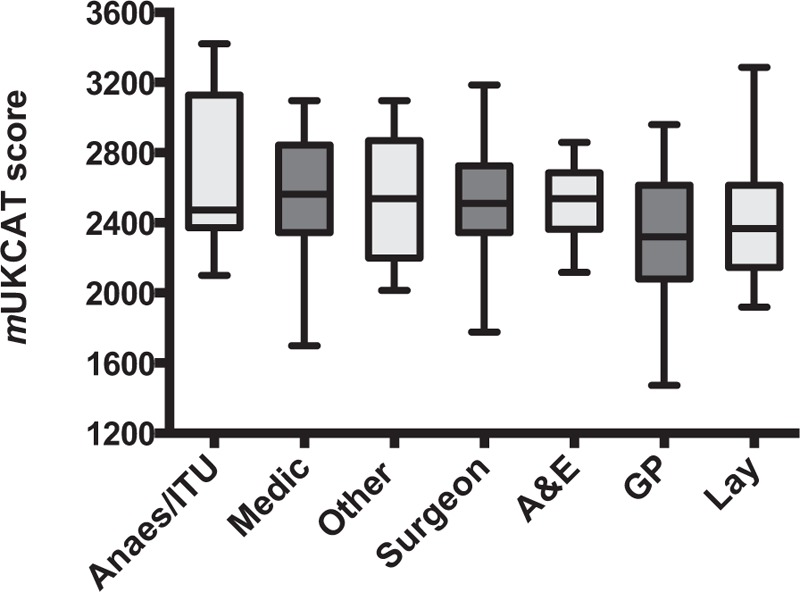
Comparison of *m*UKCAT score by specialty.

**FIGURE 4:**
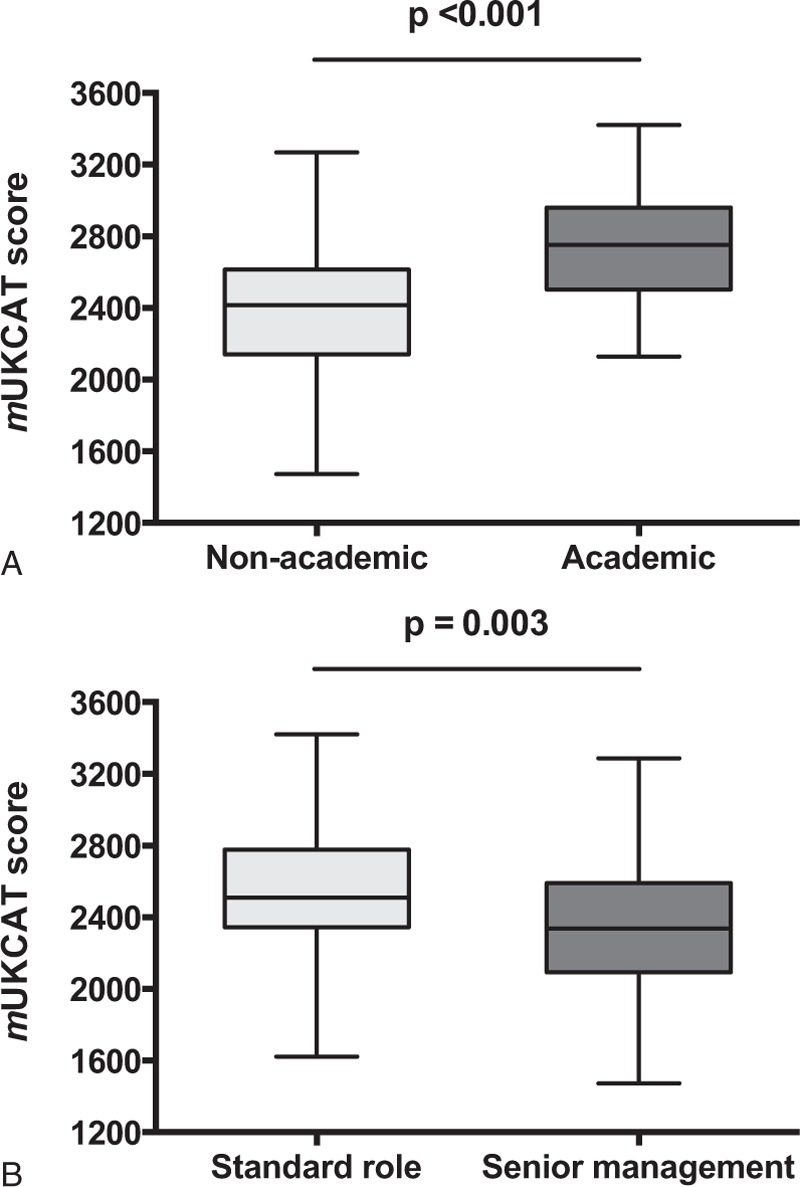
Comparison of *m*UKCAT score in clinicians by (A) academic and by (B) management status.

The overall mean *m*UKCAT score of all participants was 2486 (69.1%) (Table 1 and Figures 2–4). The spread of scores is shown in Figure 2. The highest overall score achieved in the test was by an intensivist (3421, 95.0%) and the lowest score was from a GP (1472, 40.9%). There was no significant difference in score by sex (mean score of males, n = 111 vs females, n = 82: 2491 [SD 351] vs 2479 [SD 363], mean difference 12, 95% confidence interval [CI] −90 to 114, *P* = 0.82). Medical specialty was associated with overall score (*P* = 0.003), where anesthetists and intensive care physicians scored highest and GPs lowest. Contrary to expectation, fully trained clinicians were not outperformed by doctors in training (hospital consultants [including professors] and GPs combined, mean score 2501 [SD 365] vs trainee 2483 [SD 308], mean difference 18, 95% CI −145 to 108, *P* = 0.78).

**TABLE 1:**
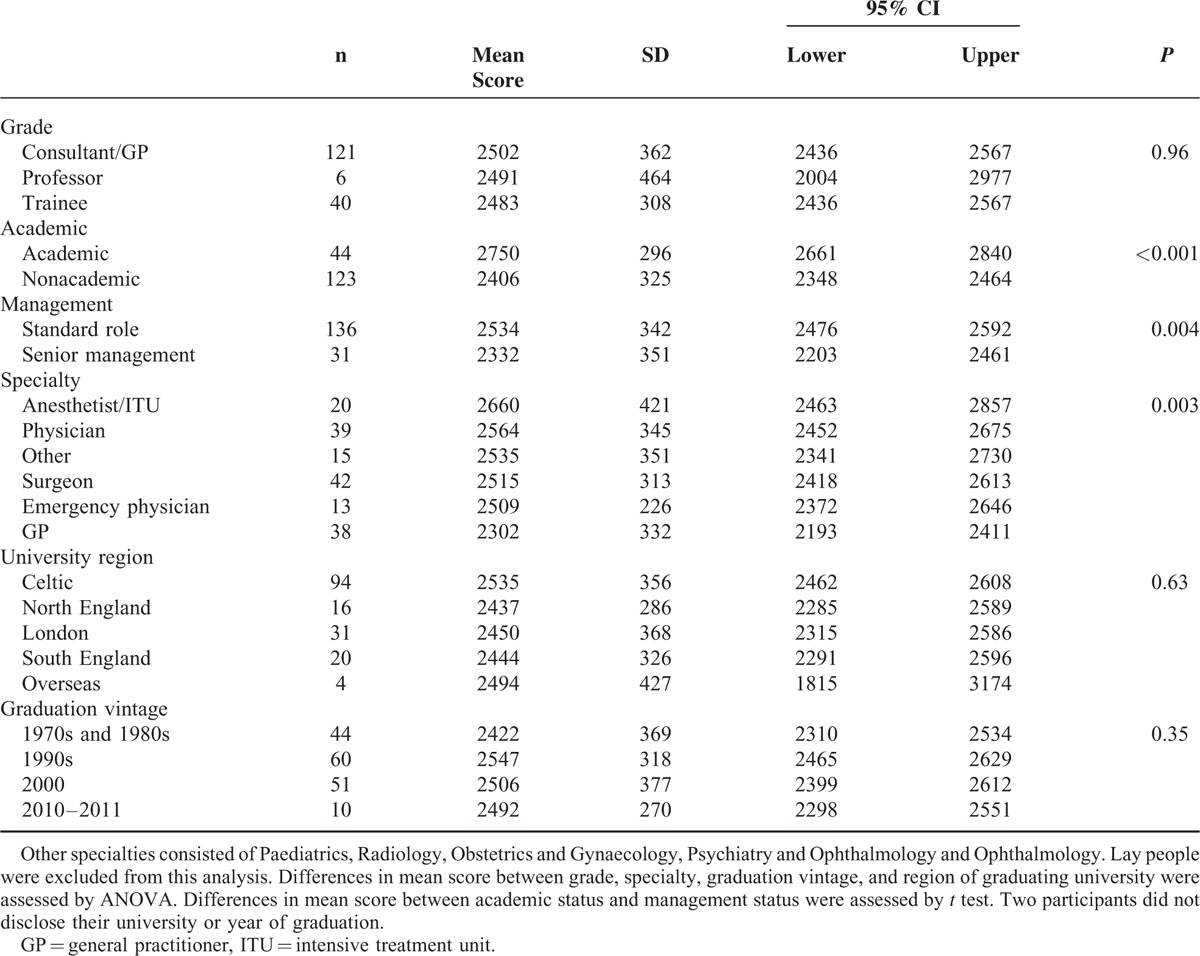
Mean score, Standard Deviation (SD) and 95% Confidence Interval (CI) by Baseline Characteristics of Clinicians

Academics performed better than nonacademics (lay people excluded, mean difference 345, 95% CI 234–455, *P* < 0.001). However, those clinicians with senior management positions scored lower than those in “standard” roles (lay people excluded, mean difference 202, 95% CI 67–337, *P* = 0.004). There was no significant difference in score by graduation vintage (*P* = 0.35) or by the geographical location of medical school (*P* = 0.63).

There was no significant difference in score between males and females (male mean score 2491 [SD 351] vs female 2479 [SD 363], mean difference 12, 95% CI −90 to 114, *P* = 0.82). There was also no significant difference in score between doctors and lay people (doctor mean score 2497 [SD 351] vs lay people 2415 [SD 380], mean difference 81, 95% CI −240 to 67, *P* = 0.28).

### Situational Judgment Section

The highest achieving individual in this section of the *m*UKCAT was a consultant general surgeon (100%). The lowest score was achieved jointly by an emergency physician in training and a lay person (investment banker 27.3%). There was no evidence that specialty was associated with the score achieved in this domain of the test (Table 2; *P* = 0.15). Clinical seniority also had no impact on situational judgment (professor 69.7%, hospital consultant or GP 65.1%, doctor in training 65.0%; *P* = 0.75). There was no significant difference in situational judgment between doctors and lay people (doctors mean 65.2% [SD 14.7] vs lay people mean 63.6% [SD 13.1], mean difference −1.58, 95% CI −7.60 to 4.44, *P* = 0.61).

**TABLE 2:**
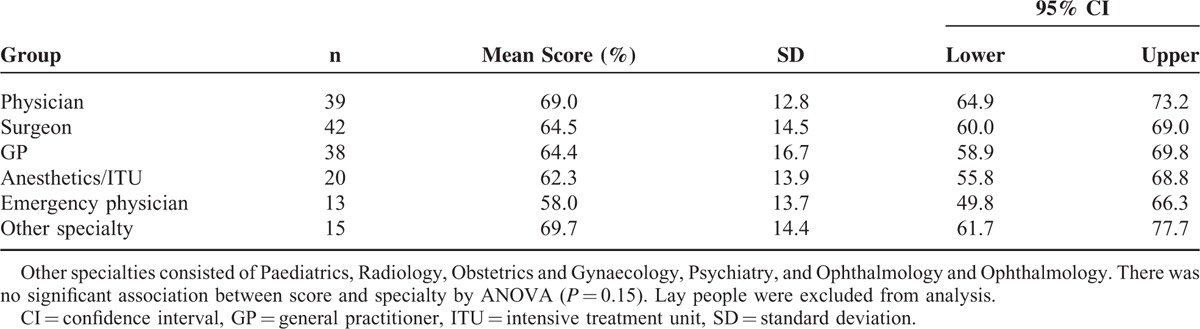
Situational Judgment Score by Specialty

For clinicians alone, those individuals in senior management positions did not exhibit greater situational judgment than their colleagues in standard roles (senior management vs standard role: 65.6% [SD 14.0] vs 63.6 [SD 17.4], *P* = 0.51). Academics exhibited greater situational judgment than their nonacademic colleagues (academics vs nonacademics: 69.8 [SD 14.5] vs 63.6% [SD 14.4], *P* = 0.01).

We received many interesting comments from participants in the study. A selection of these is included in the supplementary information. They provide some reflections on the *m*UKCAT and the use of *r*UKCAT in application to medical school.

## DISCUSSION

To our knowledge, this is the first study that examines the performance of senior clinicians in the widely used UKCAT. We have shown that the majority of currently practising senior clinicians passed our *m*UKCAT. Academics achieved higher scores than nonacademics. Anesthetists and intensive care physicians scored highest and GPs the lowest.

The UKCAT has no curriculum content and is designed to probe innate skills. It may be argued that these are gradually lost with age/seniority, as one accrues medical experience. However, the UKCAT is designed to test those abilities, attitudes, and behavior patterns that are considered to be essential for a successful medical (and dentistry) career and as such would be expected to remain present throughout a career. These attributes are of course necessary in other professions, and in keeping with this our *m*UKCAT did not select between medics and lay people.

It is not clear why some specialties outperformed others. It is possible that a deep understanding of one specialty area confers advantages over those with a more broad-based knowledge (eg, GPs), or perhaps those regularly undertaking calculations in their daily work (eg, anesthetists, academics) had some advantage. Reassuringly, clinicians performed well in the situational judgment component of the test.

The UKCAT has now entered its 10th year. The first cohort of qualified doctors who entered medical school through the UKCAT system is yet to reach senior clinician status. It will be of interest to see if passing the UKCAT as a prerequisite to an application for medicine changes the UK medical workforce, and this should certainly be an area of future research. Given the widespread use of the UKCAT in determining entry to UK medical schools, it is somewhat surprising that this has not previously been assessed. Perhaps tomorrow's doctors will be better problem solvers than their predecessors, but this might come at the expense of better communication skills. Ultimately, the crucial issue is whether the reverse phenomenon from that assessed in this study is true, that is, is the *r*UKCAT selecting medical students who are well suited to a career in anesthetics/intensive treatment unit or academia in preference to those who are well suited to a career in general practice or senior management? If true, the UKCAT may change the characteristics of the medical workforce of the future. This should be the subject of future larger-scale studies.

We recognize the difficulties facing medical schools in selecting those candidates best suited to a career in medicine. For one, there is a significant excess of applicants for the number of places available. For example, at the University of Edinburgh, this ratio is 14:1 for UK/EU students.^16^ There are some measures that help in these decisions. McManus et al^17^ have previously reported on school students’ performance at A-level as predictors of future medical careers. They showed A-level results related to performance in undergraduate medical exams, time taken to achieve postgraduate qualifications (membership/fellowships, diplomas, higher academic degrees), and also the number of research publications. Interestingly, those doctors who were no longer on the medical register were more likely to have lower A-level grades. In support of that study, a meta-analysis has shown A-levels to be a better predictor of medical school performance than the UKCAT.^18^ However, as many potential applicants to undergraduate medicine attain similarly top-level grades at A-level, we appreciate that there has to be some other way to discriminate between candidates. Objective structured clinical examination (OSCE)-style interviews are time-consuming and evidence of their validity is sparse.^19^ Proponents of aptitude tests have argued that they can identify raw talent independent of education, and in doing so widen access to medical school.^20^

Many of those participating in our study questioned whether performance could be improved with practice. Although there are no published studies examining how practice affects score, the UKCAT website reports that use of their online practice tests and use of books relevant to UKCAT are associated with higher overall performance (although this effect is not quantified).^12^ The British Medical Association (BMA) offers online revision practice questions for £26-41 for 1-3 months’ access.^21^ More in-depth courses are also available, and also individual tuition which can cost up to £1750.^22,23^ Thus, one may question the UKCAT by lines of “fairness” and “wider access.”^24^

### Strengths and Weaknesses of the Study

The strengths of this study are the range of specialties represented, the use of typical UKCAT questions, and the statistical modeling of the results to reflect the *r*UKCAT. The full *r*UKCAT test lasts 2 hours, whereas our *m*UKCAT lasted 15 minutes, with 3 minutes per domain. The length of our modified test was based on achieving a balance between validity and convenience. The study was devised to be hypothesis-generating for future more robust studies. Although the length of our test reduces its predictive validity, a lengthier test was likely to be prohibitive to clinicians taking part. Tests were not undertaken under examination conditions and we relied on self-reporting of the length of time taken. Ensuring examination conditions would have limited the study to the hospitals in which the authors worked and would likely have markedly reduced the number of participants. Responses were voluntary and hence our sample may not be representative of the wider medical workforce.

## CONCLUSIONS

The majority of clinicians passed our *m*UKCAT. Academics and anesthetists were found to be the top performers, with GPs and those in senior management positions the worst. It will be of great interest to see what sort of doctors are created by using the UKCAT and this should be the focus of bigger and better studies. Furthermore, we should be prepared to modify the test if it has unwanted consequences.

## Supplementary Material

Supplemental Digital Content
