# Supplementary material for: A Cross-sectional Study of Current Doctors’ Performance in a Modified Version of a Medical School Admission Aptitude Test: The UKCAT
Source: Medicine (Baltimore). 2016 May 6;95(18):e3506. doi: 10.1097/MD.0000000000003506 (PMC4863766; doi:10.1097/MD.0000000000003506)
Supplement: Supplemental Digital Content [file medi-95-e3506-s001.doc]

**A cross-sectional study of current doctors’ performance in a modified version of a medical school admission aptitude test, the UKCAT**

***Supplementary Information***

James P Blackmur*

Nazir I Lone^†^

Oliver D Stone^§^

David J Webb*

Neeraj Dhaun*

*British Heart Foundation Centre of Research Excellence, University of Edinburgh

^†^ Usher Institute of Population Health Sciences and Informatics, University of Edinburgh

^§^ Department of Orthopaedics, Royal Infirmary of Edinburgh

Correspondence to: Dr Neeraj Dhaun

The Queen’s Medical Research Institute

47 Little France Crescent

Edinburgh

EH16 4TJ

Telephone: (+44)-131-242-6786

E-mail: [bean.dhaun@ed.ac.uk](mailto:bean.dhaun@ed.ac.uk)

**I. The UKCAT threshold score for successful application to medical school differs for each university and on each admission cycle and is dependent on the scores of all those applying. We considered that the best proxy for a successful *m*UKCAT score was to look at the medical schools that publish their recent successful applicants’ *r*UKCAT scores. These are listed below.**

**(Available at:**

[http://www.ukcat.ac.uk/App_Media/uploads/pdf/How%20the%20UKCAT%20is%20used%202013.pdf](http://www.ukcat.ac.uk/App_Media/uploads/pdf/How the UKCAT is used 2013.pdf))

Aberdeen University

*‘Lowest total score of an applicant was 1619. The lowest score for successful applicants was 2210 points’*

We therefore took 2210 as the cut-off for successful application.

Dundee University

*‘We interviewed few applicants with a UKCAT score below 2,300 and the average for those gaining offers was over 2,600’*

We therefore took 2300 as the cut-off for successful application.

Hull-York Medical School

‘*If you have a total UKCAT score of less than 2400, or a score of less than 450 in any of the four cognitive subtests, we won't normally consider your application’*

We therefore took 2400 as the cut-off for successful application.

Imperial University

‘*For 2013 entry, 640 was the minimum score required in each of the following: quantitative reasoning, decision analysis, verbal reasoning, abstract reasoning’*

We therefore took 2560 as the cut-off for successful application.

Keele University

‘*Applicants receiving offers for 2012 entry had UKCAT total scores ranging from 2020 to 3070’*

We therefore took 2020 as the cut-off for successful application.

Leicester University

‘*Academic ability and UKCAT will each be scored out of 30, giving a total score out of 60. UKCAT will be scored according to the total, as follows: 3000+ =30, 2900+ =29, 2800+ =28, 2700+ =27 etc. Candidates achieving a UKCAT total of less than 2,100 will not be excluded, but will generate a score of zero for this section’*

We therefore took 2100 as the cut-off for successful application.

Manchester University

‘*The following tables give the range of UKCAT scores for applicants who were invited to interview:*

| ***Year*** | ***Successful score*** |
| --- | --- |
| 2012/13 | 2650 and above |
| 2011/12 | 2590 and above |
| 2010/11 | 2610 and above |

We took the minimum of these, 2590, as the cut-off for successful application.

Newcastle University

*‘Successful applicants should score “not less than 550 in any individual component”’*

We therefore took 2200 as the cut-off for successful application.

Queen's University London

‘*It is unlikely that you would be offered an interview if you obtained a TOTAL UKCAT score below 2400’*

St.George's London

‘*The minimum overall score for 2013 entry to the Medicine MBBS course is 2530’*

Southampton University

‘*Applicants “must score 2500 or above in the UKCAT exam in order for their application to be considered further”’*

Warwick University

‘*We are unlikely to invite anyone with a UKCAT total score that is below 2600 to the Selection Centre process’*

We took the mean of these scores (2368) as our cut-off for a ‘successful’ *m*UKCAT score. Those scoring above this level were deemed to have ‘passed’ our *m*UKCAT.

**II. Throughout the study period we received many comments from participants. To demonstrate the general appeal of our study across the broad range of medical specialties, and to explain the background to some of the discussion points, we have included some of these below:**

**I. Some participants thought the test was strange and many questioned the utility of the actual UKCAT in selecting potential doctors:**

*“I thought it was very hard and couldn’t do the sets at all but liked the maths. It really is quite a bizarre test and am not sure at all picks out suitable doctors, where is the empathy, sensitivity etc which are more important than ability to recognise patterns and sets in my opinion!”*

***Consultant Haematologist***

*“I am not sure how the test really relates to being a doctor. Look forward to seeing your final conclusions.”*

***Consultant Geriatrician***

*“Bizarre, bizarre test. The very first question stumped me for ages...and I still ended up guessing. Not enough information... moan, moan, moan. I don't know how rhomboids affect my abilities as a Dr either??? I look forward to hearing your results when they get published!”*

***GP & RCGP Examiner***

*“I have to say I found the picture round completely baffling. Good to know that being able to line up triangles will make me a better doctor…I get the feeling it is the sort of test you could get quicker/better at with practice…Good luck with the research – nice to see someone challenging the medical educationalists at their own game.”*

***Consultant Paediatrician***

*“[In the test], our conclusion is that to get the best result the solution is to rush everything, guess and avoid the difficult questions! This would be a sad indication of how to become a doctor.”*

***General Practitioner***

*“How any of that relates to medicine is beyond me.”*

***Consultant Transplant Surgeon***

*“It is very interesting. Some of that stuff was gobbledigook to me…… I continually feel that a significant proportion of the young doctors lack the attitude and or aptitude to develop multiple skills and take responsibility, often being fantastic at collecting information and able to demonstrate fantastic knowledge about conditions but struggle to put the two together to make a diagnosis and management. ………. The present system to me is a bit like buying a horse, you measure everything and end up with a perfect horse, but you have never assessed whether it can run.“*

***GP Partner and Trainer***

*“The wrong people are getting into medicine. We don’t need them all to be very clever, we need those who are willing to work hard and are very practical… When completed would you present you work?”*

***Consultant Anaesthetist***

*“There is concern that selection processes into medical school…are not identifying potential family doctors but instead choose those with the desire and skills to become academics and neurosurgeons...”*

***GP Partner and Trainer***

*“[The tests are] a bit like IQ tests –the candidates practice a lot and it largely demonstrates that you’re good at doing these sort of tests – extrapolation to career choice may be tenuous. One of the great things about medicine is that it is so broad, there is a place for everybody, for Asperger’s, introverts, extroverts, the practical, the abstract thinker, the loner, the gregarious etc. etc. We all found a place, whether in lab research, orthopaedics, psychiatry, Medecin sans Frontiere, radiologist, paediatric haemato-oncologist, occupational health, public health, the Department of Health. Is using one tool to sieve the applicants casting the net too narrowly (to mix two metaphors)? Are we turning down some eminently suitable candidates? Are we selecting the wrong people with it? The dropout rate seems to be much higher (both pre-and post registration) than it was 25 years ago. Or maybe we really are more **** at the job than we care to admit!”*

***Consultant Anaesthetist***

**II. Others simply found the study an interesting discussion point:**

*“Thanks so much for including me in your study...it's started many an interesting debate amongst our social circle, and we will all await your findings when they get published.”* ***Psychologist***

*“As a parent of an 18 year old we have been talking about this very subject by the side of the school rugby pitch.”*

***Consultant ENT Surgeon***

**III. Some commented on the cost of revision courses:**

*“Interestingly revision courses cost £1-2k making it a good way to increase accessibility to med school!”*

***General Surgical Registrar***

**IV. And most were just glad they did not have to sit the test when they applied to medical school:**

*“Poor souls that have to do that to get into uni!”*

***General Practitioner***

*“I am not sure if I am happy or sad that I have passed (barely) this wee test. I would certainly be a grumpy person if my whole future career was based on the result of this sort of nonsense!!!!!  Obviously any profiling test that I do not score 100% in cannot be validated as I am the best therefore by definition should be getting it all right.  Please include this fact in your overall analysis.”*

***Consultant Urologist***

*“I found the abstract reasoning the most difficult to fathom. I am glad this wasn’t part of the selection process when I applied in the distant past in the 1980s.”*

***Consultant Geriatrician***

*“Glad I was a doc before this kind of stuff!”*

***Consultant Psychiatrist***

**V. Finally, a few seemed moved to either rage or self-harm!**

*“My nose is bleeding after this!”*

***Consultant ENT Surgeon***

*“Words cannot describe how angry this makes me - I am very glad I sneaked into medical school before these things existed.”*

***Consultant Radiologist***
